# Efficient Volumetric Absorption Solar Thermal Platforms Employing Thermally Stable - Solar Selective Nanofluids Engineered from Used Engine Oil

**DOI:** 10.1038/s41598-019-47126-3

**Published:** 2019-07-22

**Authors:** Nirmal Singh, Vikrant Khullar

**Affiliations:** Mechanical Engineering Department, Thapar Institute of Engineering and Technology, Patiala, 147004 Punjab India

**Keywords:** Solar thermal energy, Nanoparticles

## Abstract

We report a low cost and scalable method to synthesize solar selective nanofluids from ‘used engine oil’. The as-prepared nanofluids exhibit excellent long-term stability (presently tested up to 6 months under undisturbed stagnant conditions at room temperature) and photo-thermal conversion efficiency. Moreover, these were found to retain their stability and functional characteristics even after extended periods (72 hours) of high temperature (300°C) heating, ultra violet light exposure and thermal cyclic loading. Building upon it, we have been able to successfully engineer an efficient volumetric absorption solar thermal platform that employs the as-prepared nanofluids and achieves higher steady state temperatures (approximately 5% higher) relative to the conventional surface absorption based solar thermal system under the sun. The developed volumetric absorption solar thermal platform could prove to be significant step in the evolution of efficient solar thermal systems which could potentially be deployed for host of applications ranging from solar driven heating, air-conditioning, and desalination units to solar energy electricity generation systems.

## Introduction

Nature has carefully engineered itself to utilize solar energy to run the entire life cycle on the planet earth. Mimicking natural processes, we are continuously striving to build efficient solar energy conversion platforms which could convert solar energy to more usable forms such as electricity (photovoltaic), chemical energy of the fuel (artificial photosynthesis) and heat (solar thermal). Amongst these, the technology of electricity generation via solar to heat conversion is currently the most efficient (~30%) and cost effective one. In comparison, the fossil-fuel based counterparts operate at efficiencies on the order of ~60%. One of the key reasons for lower efficiency is that we have not been able to efficiently engineer the solar (photon) to thermal energy (heat) conversion process^1^.

With the advent of nanotechnology, plasmonic nanostructures (particularly metallic nanoparticles) have evolved as potential candidates for efficient photo-thermal conversion at their resonant frequencies owing to predominant non-radiative decay of the absorbed energy in the form of heat^2–4^. However, sunlight being broad spectrum necessitates plasmonic nanostructures (such as carbon-based nanostructures) that could respond to the wide wavelength range of incident sunlight^5–7^.

Building upon the idea of utilizing nanostructures for solar to thermal energy conversion process; volumetrically absorbing solar thermal systems employing nanoparticle dispersions (nanofluids) have been devised by various researchers. Theoretically (and on laboratory scale), particularly at high solar concentration ratios (solar flux), the nanofluid-based volumetrically absorbing solar thermal systems have been shown to have higher thermal efficiencies, lower embodied energies and lower carbon footprints relative to their surface absorption based counterparts^8–11^. However, these promising novel systems have not been able to outperform the incumbent solar thermal platforms under the sun owing to inefficient receiver designs and instability of nanofluids in real world service conditions - nanoparticles tend to agglomerate and hence settle down; this drastically affects the optical efficiency and hence the overall performance of these systems^12,13^.

Presently, a lot of efforts are underway to tailor solar selective, low cost, high temperature, and long term stable nanoparticle dispersions^14–30^. In this direction, we propose that ‘used engine oil’ (owing to the presence of carbon soot particles) could be employed to synthesize broad wavelength absorption nanoparticle dispersions (volumetrically absorbing solar selective heat transfer fluid).

Annually, approximately 24 million metric tons of ‘used engine oil’ is discharged into the environment without any recycling or treatment^31^. Therefore, forming one of the most hazardous wastes; having irreversible environmental and health implications. Putting this otherwise hazardous waste to harness solar energy could prove to be a sustainable option.

Pristine (or un-used) engine oil essentially consists of base oil (or blend of base oils) and an additive package to enhance its anti-oxidant, anti-wear, anti-foaming, and dispersancy characteristics. During its operation, the engine oil comes in contact with high temperature cylinder liners and washes away the carbon soot particles (left after combustion) from the cylinder circumference. Furthermore, a host of metallic particles (as a result of wearing action) enter the engine oil. The blowby gases also enter the crankcase which may tend to oxidize the engine oil; and here comes the role of anti oxidant which interrupts the oxidation mechanism by reacting with the reaction intermediates^32–36^. The presence of dispersant molecules helps in dispersing the aforementioned foreign particles in the oil by forming an envelope around these particles. The polar part of the dispersant molecule attaches itself to the surface of the particle; whereas the oleophilic long chain hydrocarbon part helps in mobility of the particle. This ensures that the soot particles do not interact with each other and hence prevents agglomeration of the soot particles; i.e.; agglomeration is prevented through steric stabilization^33^. Now, the effectiveness of dispersant in dispersing the soot particles depends on the effective reactive surface area available on the soot particle where adsorption of the polar part of the dispersant could take place. This is important as many researchers have observed that certain combustion conditions result in un-reactive soot particles; and furthermore, other polar molecules in the engine oil may also get adsorbed on the soot surface and hence reducing the effective reactive surface area available for the dispersants^32,36^. In the service life of the engine oil, as the concentration of the soot particles increases; more number of soot particles compete for the available dispersant and also thermal degradation (resulting in conformal changes in the oleophilic chain of the dispersant) as well as the oxidation of the dispersant happens - this results in decrease in dispersancy of the soot particles and hence thickening of the engine oil owing to soot particles agglomeration^32,36^. After the end of the service life of the engine oil, in addition to resin, sludge etc.; it consists of large number of nano-sized soot particles which are still enveloped by dispersant molecules. It is the essentially the presence of these ‘sterically stabilized carbon soot particles’ in the used engine oil that qualifies it to be used as a precursor for synthesizing heat transfer fluid for direct absorption of solar energy.

Rigorous testing (which simulate real world service conditions) of the as-prepared nanofluids reveals that these have remarkable photo-thermal conversion efficiency, favorable thermo-physical properties (thermal conductivity and viscosity), high temperature and long term stability, and can withstand thermal cyclic loads without any significant loss of optical and thermo-physical properties. Building on it; a hybrid volumetric receiver employing the as-prepared nanoparticle dispersions has been carefully designed to give higher steady state temperatures (and hence higher thermal efficiency) relative to the conventional surface absorption based receiver under real world outdoor conditions.

In essence, the present work is a significant step in the evolution of solar thermal platforms; wherein we have been able to design a unique volumetric absorption based receiver that employs thermally stable and solar selective nanoparticle dispersions engineered from ‘used engine oil’ and has higher efficiency relative to its surface absorption based counterpart under the sun.

## Results and Discussion

### Understanding soot dispersancy in used engine oil

In order to understand the reasons for dispersancy of soot particles in used engine oil; in addition to ‘used engine oil’, we also need to analyze two variants of soot particles extracted from the used engine oil -namely - the one in which dispersant macromolecules remain attached to the soot particles (referred to as Type-1) and the other in which there are no attached dispersant macromolecules (referred to as Type-2). Now, in order to extract the two aforementioned soot types from used engine oil, we need to understand solution thermodynamics. The interaction energy between the solvent and polymer macromolecule is given by Eq. (1) as^37^

1 $${\rm{\Delta }}\varepsilon =-K{({\delta }_{s}-{\delta }_{p})}^{2}$$

where *K* is a positive constant, and *δ*_*s*_ and *δ*_*p*_ are the Hildebrand solubility parameters of solvent and polyolefin (polymer macromolecule) respectively. A value Δ*ε* near zero [in other words, low magnitude of (*δ*_*s*_ − *δ*_*p*_)] signify good solubility; whereas, values away from zero [high magnitude of (*δ*_*s*_ − *δ*_*p*_)] signify poor solubility^37–39^.

Firstly, in order to extract Type-1 soot particles, we use 1-butanol (3:1), an extraction-flocculent solvent, which through it anti-solvent action on the polymeric macromolecules results in co-flocculation of soot and polymeric soot particles together. This may be attributed to the high value of (*δ*_*s*_ − *δ*_*p*_), for 1-butanol as the solvent.

Now, in order to extract Type-2 soot particles, we follow the same procedure as followed in extraction of Type-1 soot particles, followed by treatment with a hydrocarbon based solvent (n-heptane in the present work). Here, Type-1 soot particles are mixed in n-heptane followed by centrifugation (for 10 minutes @8000 rpm) - resulting in removal of oil as well as dispersant macromolecules from the carbon soot particles [the value of (*δ*_*s*_ − *δ*_*p*_) being small with n-heptane as the solvent)^38,39^.

**Figure 1:**
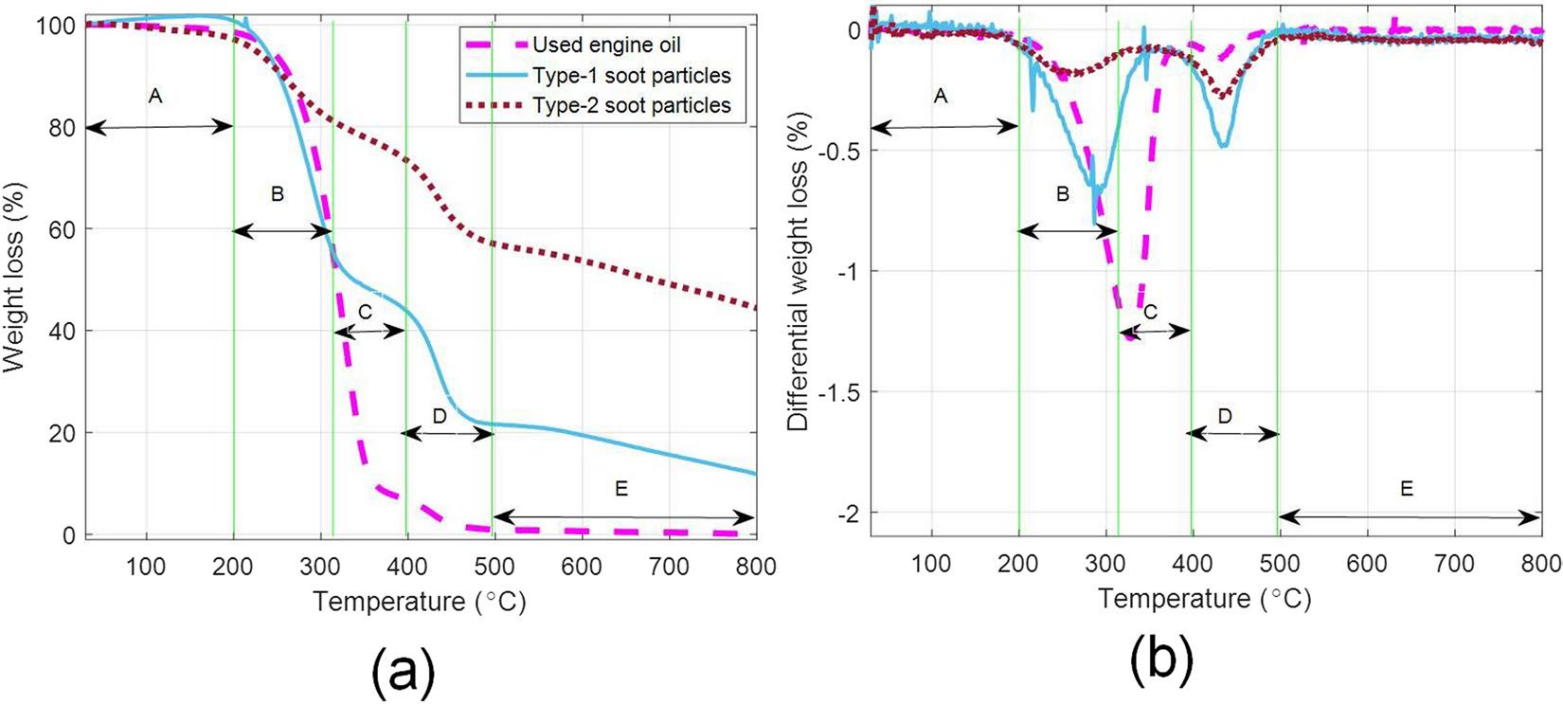
TGA: Weight (%) loss and (**b**) differential weight loss as a function of temperature for used engine oil, Type-1 soot particles and Type-2 soot particles.

Finally, Thermo-gravimetric analysis (TGA) was carried out on all the three samples namely- ‘used engine oil’, ‘Type-1 soot particles’, and ‘Type-2 soot particles’. Figure 1(a,b) show weight loss (%) and differential weight loss (%) as function of temperature. Clearly five distinct regions could be identified in these plots [marked as regions ‘A’ (30°C–200°C), ‘B’ (200°C–310°C), ‘C’ (310°C–400°C), ‘D’ (400°C–500°C), and ‘E’ (500°C–800°C)]. Region ‘A’ depicting no appreciable weight loss, region ‘B’ showing weight loss due to evaporation of light hydrocarbons, region ‘C’ depicting weight loss due to evaporation of relatively heavy hydrocarbons, region ‘D’ depicting decomposition/desorption of the attached dispersant molecules and region ‘E’ represents the pyrolysis of the carbon soot particles. Here, the region ‘D’ is of particular interest as it represents the quantum of dispersant macromolecular-soot particles interactions - being highest in the Type-1 soot particles - confirming that indeed dispersant macromolecules are adsorbed on the soot particles.

Furthermore, when two soot particle types (Type-1 and Type-2) were separately dissolved into paraffin oil; Type-1 soot particles showed complete miscibility in paraffin oil. Complete reversal of flocculation is a characteristic of sterically stabalized dispersion^40^. Hence, confirming that soot particles are sterically stabalized in engine oil.

However, in case of Type-2 soot particles, partial miscibility was observed - particles separating out of the solution. The partial miscibility could be attributed to incomplete removal of oil/dispersant macromolecules during treatment with n-heptane [this is also apparent from the TGA of Type-2 soot particles; see region D, Fig. 1(b)].

### Nanofluid synthesis philosophy and elemental-morphological analysis

**Figure 2:**
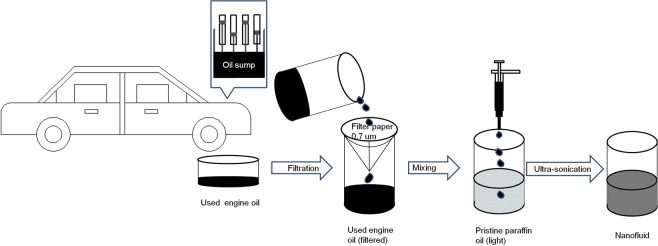
Schematic showing the steps involved in nanoparticle dispersion synthesis.

In the present work, small fractions of ‘used engine oil’ (after undergoing filtration process) have been mechanically mixed with compatible non polar base oil (paraffin oil light). The mixture was then ultra-sonicated to get the required nanoparticle dispersions (see Fig. 2).

**Figure 3:**
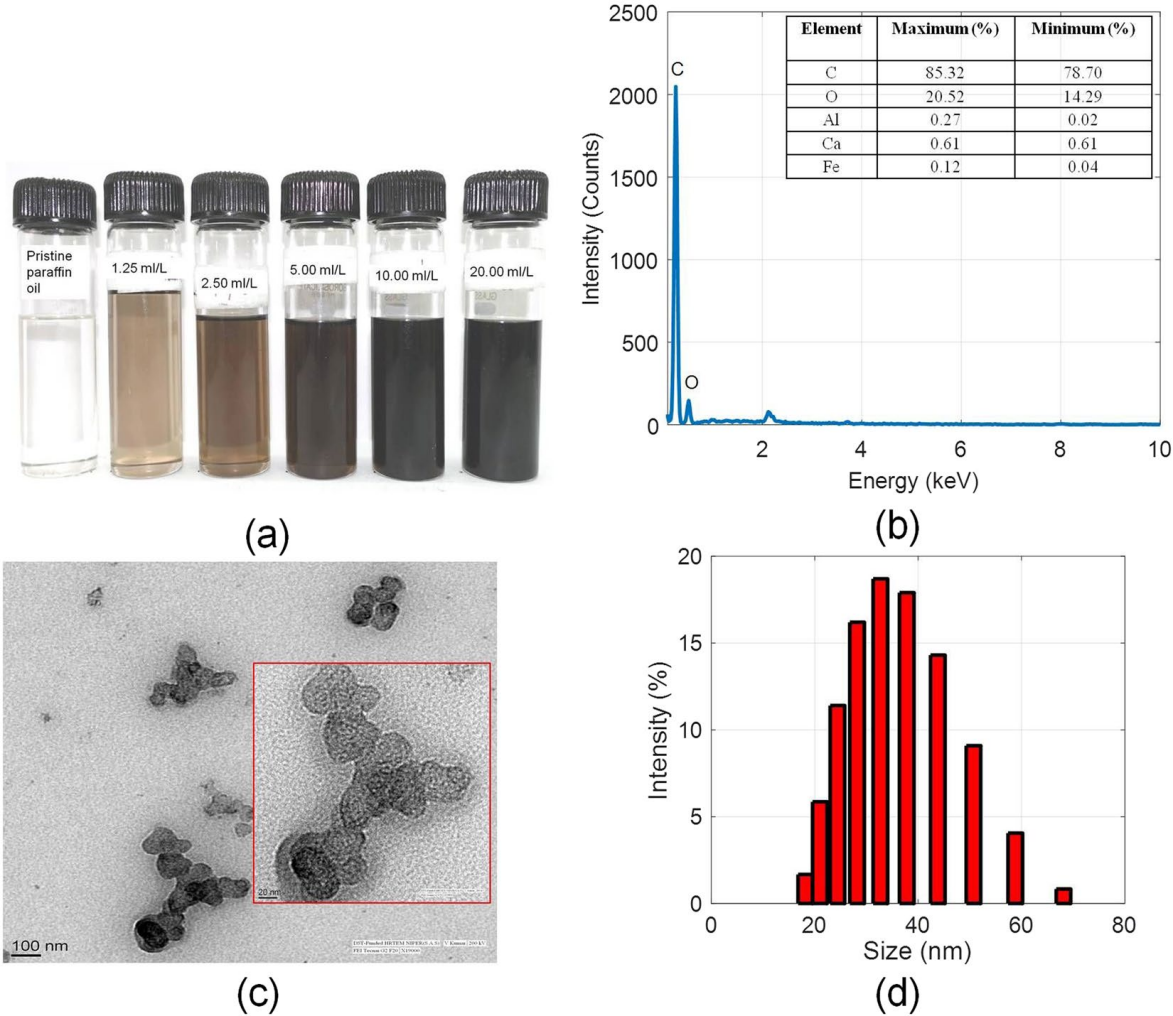
(**a**) Photographs of the as-prepared nanofluids of different concentrations, (**b**) EDS of the residue left after evaporating 20 ml/liter nanofluid sample, (**c**) TEM images of the soot particles in the used engine oil, and (**d**) Dynamic light scattering (DLS) measurements of the as-prepared nanofluid sample (5 ml/L).

Figure 3(a) shows the picture of as-prepared nanofluids of different concentrations (1.25, 2.5, 5, 10 and 20 ml/L) of ‘used engine oil’ in pristine paraffin oil. As shown in Fig. 3(b), through Energy-dispersive X-ray spectroscopy (EDS), the percentage of carbon soot particles extracted from the as-prepared nanoparticle dispersion has been found to be on the order of ~85% by weight (other notable elements being O, Al, Ca, and Fe). Transmission electron microscopy (TEM) images show that the soot particles are present in the form of nano-clusters of irregular curvilinear geometry [see Fig. 3(c)]. Hydrodynamic particle size has been measured through DLS; particle size varies in the range of 15 nm to 68 nm, average particle size being 38 nm [see Fig. 3(d)].

### Photo-thermal conversion efficiency

**Figure 4:**
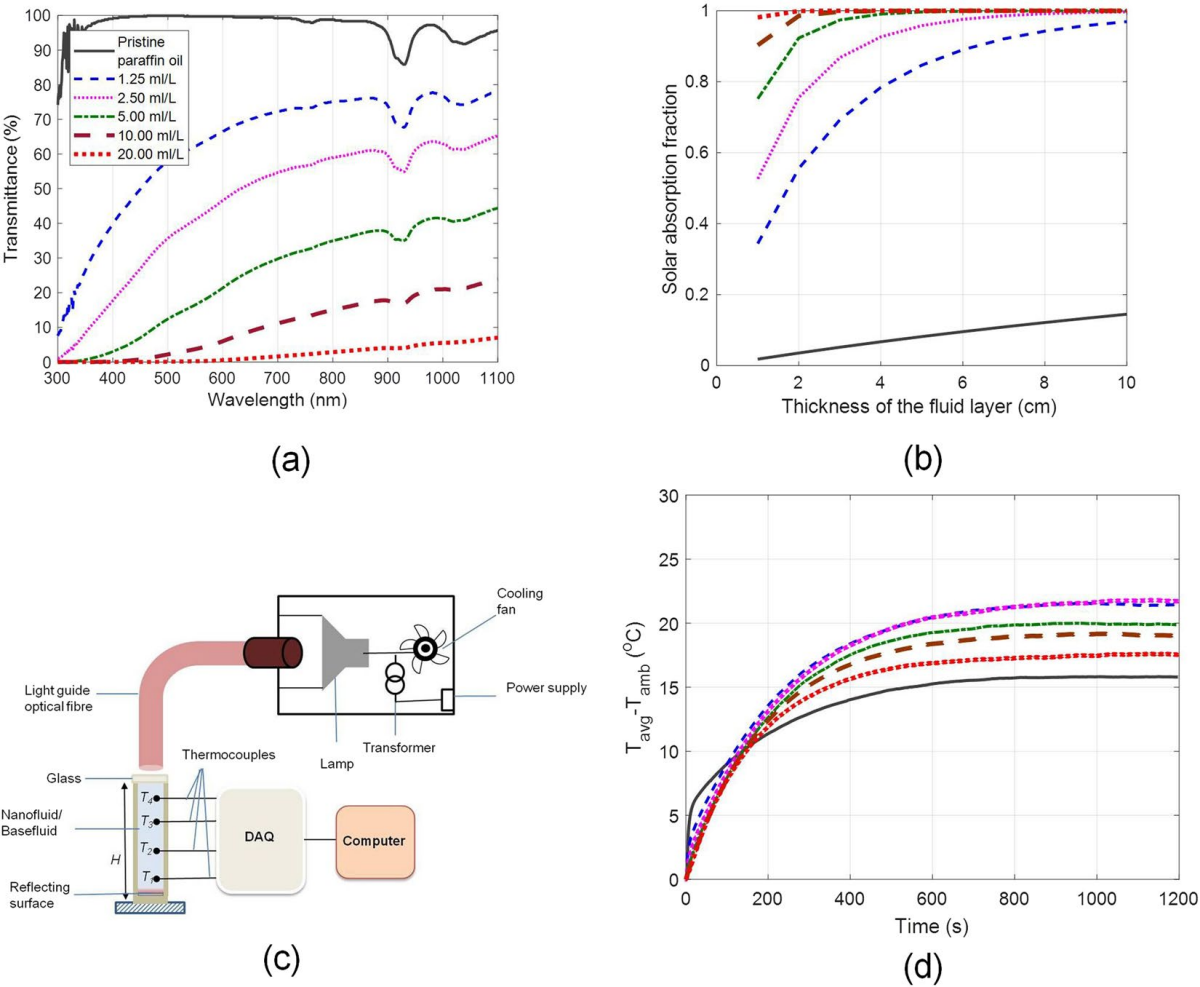
(**a**) Spectral transmittance of the as-prepared nanofluids in the UV-VIS-NIR region, (**b**) Solar absorption fraction for various nanofluid concentrations as a function of fluid layer thickness, (**c**) schematic showing the experimental set-up for photo-thermal conversion experiments, and (**d**) steady state temperatures for various concentrations of nanofluids. *T*_avg_ = (*T*_1_ + *T*_2_ + *T*_3_ + *T*_4_) /4.

As a first step, optical signatures of various nanofluid concentrations have been measured in the UV-VIS-NIR region (300 nm–1100 nm). Figure 4(a) clearly shows that pure paraffin oil transmits nearly all the incident radiation whereas 20 ml/L nanofluid absorbs nearly in the entire wavelength band; thus giving us a fair idea about the absorption capability of the as-prepared nanofluids. This data was then employed to calculate the solar absorption fraction for different nanofluid concentrations as a function fluid layer thickness as given by Eq. (2)

2 $${\rm{Solar}}\,{\rm{absorption}}\,{\rm{fraction}}=\frac{{\int }_{300nm}^{1100nm}{S}_{\lambda }[1-\exp (-{K}_{a\lambda }y)]d\lambda }{{\int }_{300nm}^{1100nm}{S}_{\lambda }d\lambda },$$

where *S*_*λ*_ is the spectral solar irradiance (AM 1.5 spectra), *K*_*aλ*_ is the spectral absorption coefficient, and *y* is the fluid layer thickness. Solar absorption fraction essentially gives the fraction of the incident solar energy that could be absorbed by a given thickness of the fluid layer. Clearly, solar absorption capability increases rapidly with increase in concentration. Moreover, to achieve the desired value of solar absorption fraction, we could either increase the concentration or increase the physical thickness of the nanofluid layer - in effect increasing the optical depth.

In order to clearly gauge the photo-thermal conversion efficiency of the as-prepared nanofluids, laboratory scale experiments have been carefully designed. Nanofluids (housed in a cylindrical column with reflective surface at the bottom) of different concentrations were illuminated with a broad spectrum white light source [see Fig. 4(c)]. Samples were illuminated until these reached steady state temperatures. These measured steady state temperatures (averaged across the entire depth of the nanofluid column) essentially represent the photo-thermal conversion efficiencies of various nanofluid concentrations under optical heating. Figure 4(d) clearly points out that nanofluids have higher steady state temperatures (on the order of ~31 higher) relative to the case of pure paraffin oil. Interestingly, the highest concentration nanofluid (20 ml/L) does not have the highest photo-thermal conversion efficiency; instead it is highest for the nanofluid with a moderate concentration (2.5 ml/L). This could be understood from the spatial temperature distribution across the depth of the nanofluid column for various nanofluid concentrations (see Supplementary Fig. S1). Spatial temperature field gives us insights into the photo-thermal conversion process. For a fixed physical thickness of the fluid layer, with increase in nanoparticle concentration, the photo-thermal conversion process tends to be limited to only top layers; not allowing the light to reach the lower layers-hence resulting in lower average steady state temperatures at very high concentrations.

### Thermo-physical properties

**Figure 5:**
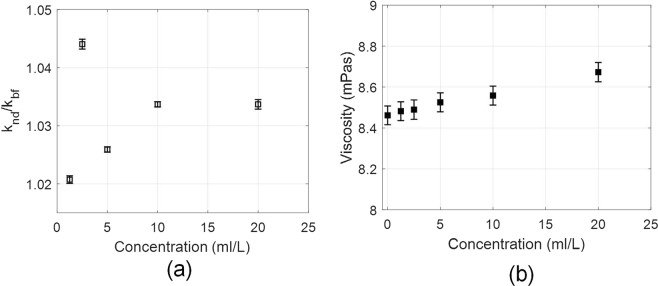
(**a**) Thermal conductivity ratio (*k*_*nd*_/*k*_*bf*_), and (**b**) kinematic viscosity as a function of nanofluid concentration. Error bar represents the standard deviation.

Thermal conductivity and viscosity are amongst the key thermo-physical properties that impact the redistribution of the absorbed energy within the fluid and pumping power requirements respectively. The as-prepared nanofluids show thermal conductivity (measured using transient hot wire method, KD2 pro) enhancements [see Fig. 5(a)] of typically 2–4% (relative to pure paraffin oil); although not a significant enhancement, but could prove to be beneficial under high solar flux conditions. Thermal conductivity enhancements may be attributed to combined effects of ballistic phonon motion, Brownian motion, thermal boundary resistance and mass difference scattering^41,42^. However, a more detailed work is required to understand the dominant mechanisms resulting in changes in thermal conductivity values. Viscosity measurements (made using capillary action viscometer) show linear increase in the viscosity with increase in concentration of the nanofluid, the increase being not greater than 2.5% [see Fig. 4(b)] even for the highest concentration (20 ml/L). Therefore, there shall not be requirement of additional pumping power when using these fluids in actual practice.

### Stability of the as-prepared nanofluids

#### Long-term stability

**Figure 6:**
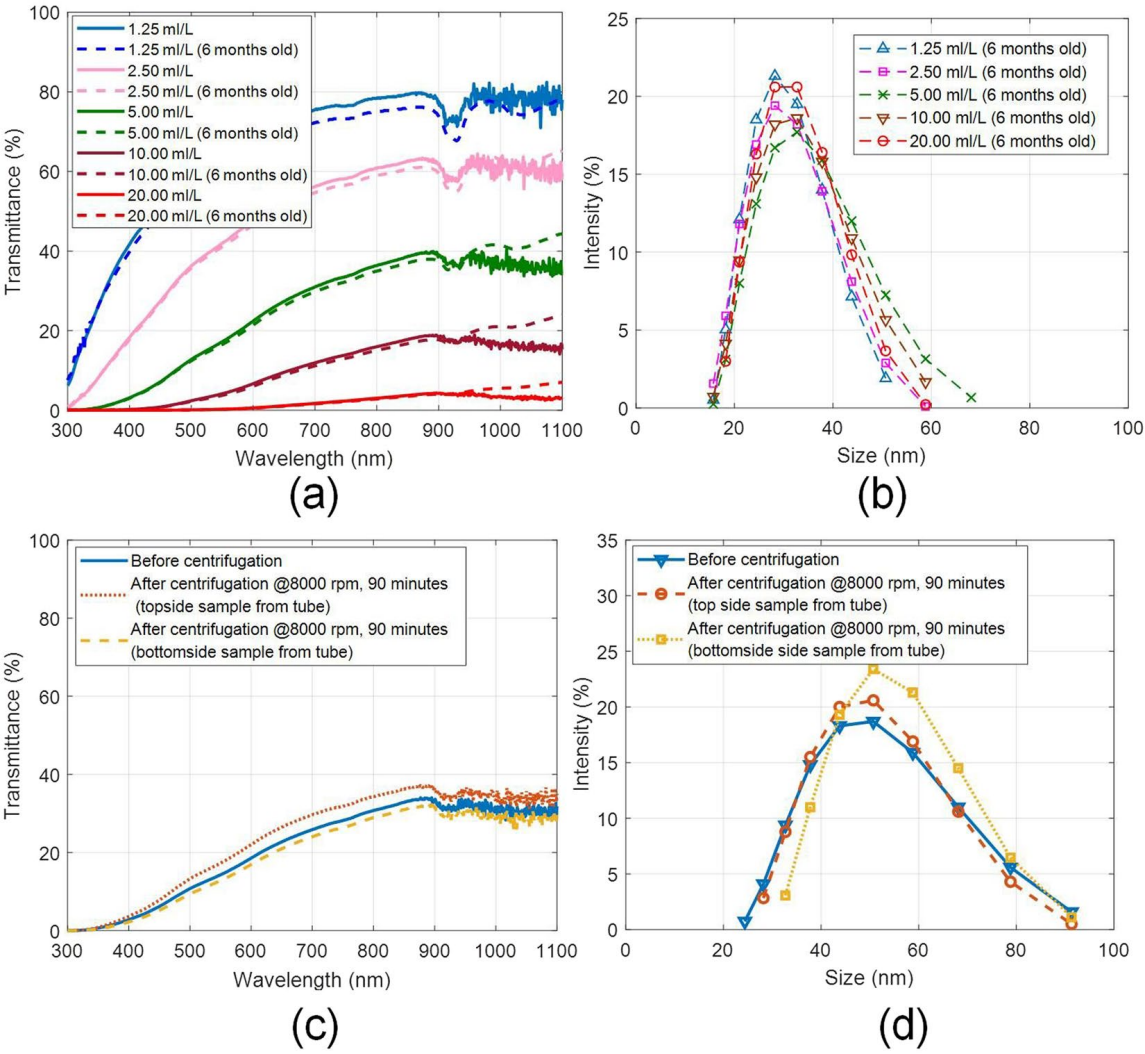
(**a**) Spectral optical characteristics, and (**b**) hydrodynamic particle size distribution of the as-prepared and six months old nanofluid samples; and (**c**) spectral optical characteristics, and (**d**) hydrodynamic particle size distribution of the as-prepared and after centrifugation (for 90 minutes @8000 rpm) nanofluid samples (5 ml/L).

As ‘heat transfer fluids’ in volumetric absorption platforms; nanofluids are expected to maintain their optical and thermo-physical properties under extended periods without any appreciable degradation for consistent and efficient photo-thermal conversion. Long-term stability of the as-prepared nanofluid dispersions has been assessed under natural and accelerated sedimentation (centrifugation) conditions. Furthermore, during centrifugation, the fluid experiences severe shear stresses^43–48^- simulating real flow conditions which the heat transfer fluid may be subjected to in actual solar thermal systems. Figure 6 reveals that the as-prepared nanofluids exhibit remarkable long term stability and can withstand high shear stresses without any appreciable change in their optical characteristics, and nanoparticle hydrodynamic size distribution.

#### High temperature stability

In addition to high photo-thermal conversion efficiency, the nanofluids should maintain their desired characteristics under constant and cyclic thermal loads. In its service life, the nanofluids are expected to absorb high solar flux (particularly in high solar concentration solar thermal systems), which in turn shall result in rapid and significant temperature rise. Furthermore, the nanofluid shall transfer the absorbed energy to a secondary fluid (such as water) - thus experiencing rapid temperature drops. These rapid heating and cooling cycles form the integral part of any power cycle in general and solar electricity generation systems (SEGS) in particular. The as-prepared nanofluids were found to posses excellent stability and retain their functional characteristics under constant as well as cyclic thermal loads. During cyclic thermal loading, the nanofluid was rapidly heated to a particular fixed temperature and then was suddenly cooled by dipping it into the water bath maintained at room temperature (see Supplementary Fig. S2). For the purpose of tracking the temperatures, K-type thermocouple remained dipped in the nanofluid during the entire testing period. This however allowed the ambient air (oxygen) to interact with the nanofluid which in effect resulted in the oxidation of the basefluid (paraffin oil, see Supplementary Fig. S3) as well as the oxidation of added used engine oil (See Supplementary Fig. S4) - proving to be detrimental to the stability of the nanofluids particularly at high temperatures (see Supplementary Figs S5 and S6). Similar phenomenon was discovered during constant thermal loading (12 hour heating at constant temperature) as well (see Supplementary Fig. S7) - indicating that it is not the thermal stresses but oxidation of the nanofluid that renders the nanofluid unstable at high temperatures. Interestingly, when nanofluid was prepared using oxidized paraffin oil as the basefluid; it was observed that the added used engine oil was not fully miscible - as clouds of used engine oil could clearly be seen (although particles did not settle out). Thus, oxidation of basefluid hampers the solubility of the used engine oil. As a whole; instability of the nanofluid happens both by oxidation of the basefluid as well as the oxidation of the dispersant macromolecule attached to the soot particle - the later being responsible for settling out of soot particles from the solution.

**Figure 7:**
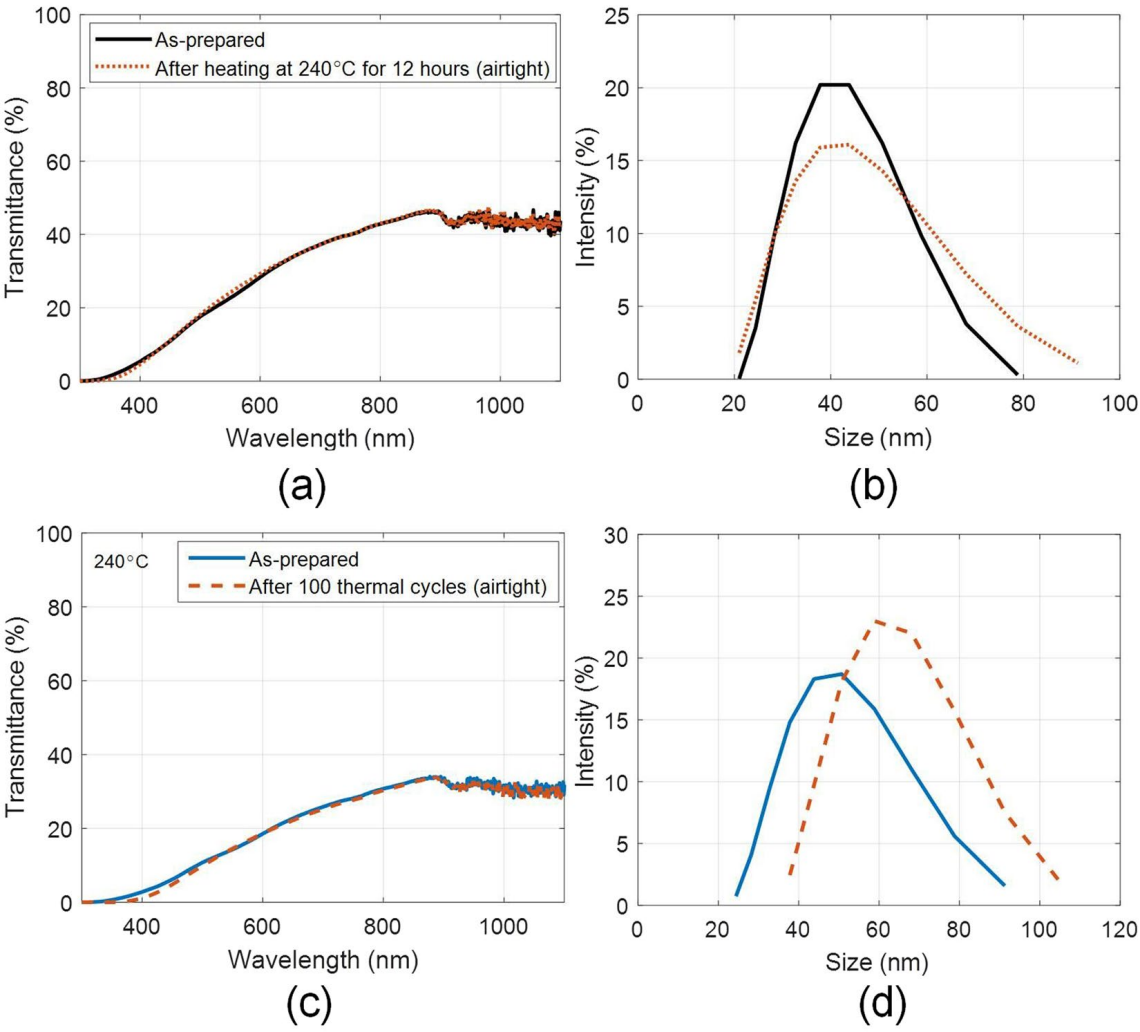
(**a**) Spectral optical characteristics, and (**b**) hydrodynamic particle size distribution of the as-prepared and after heating (for 12 hours at 240 °C) nanofluid samples; and (**c**) spectral optical characteristics, and (**d**) hydrodynamic particle size distribution of the as-prepared and after 100 thermal cycles nanofluid samples (5 ml/L); tested under airtight conditions.

Instructively, when the constant and cyclic heating tests were carried out by making the container housing the nanofluid ‘airtight’ (i.e., sealed, to ensure that no outside air enters the container); no agglomeration or settling of the nanoparticles was observed for both constant [see Fig. 7(a,b)] as well as cyclic heating tests [see Fig. 7(c,d)].

**Figure 8:**
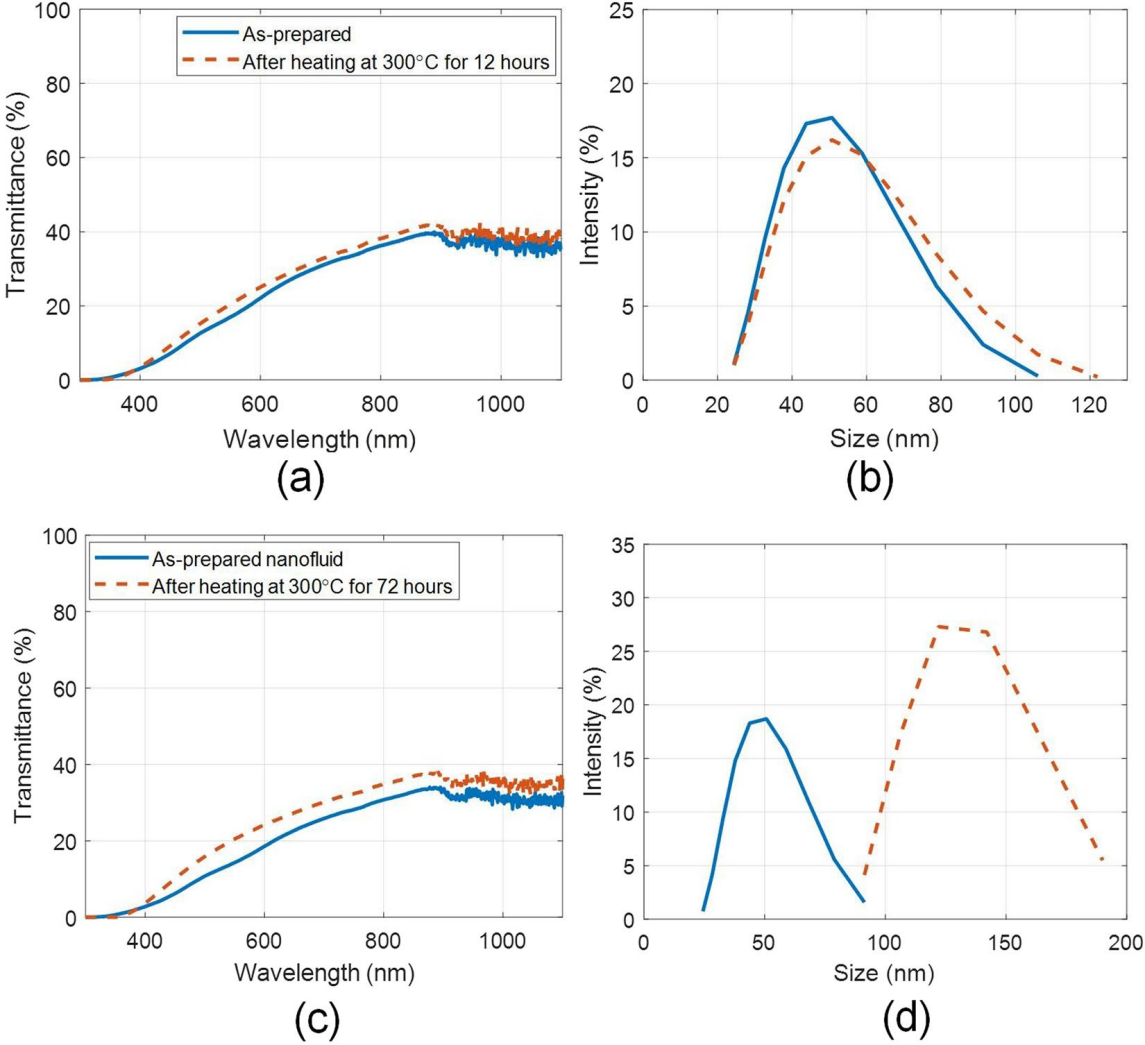
(**a**) Spectral optical characteristics, and (**b**) hydrodynamic particle size distribution of the as-prepared and after heating (for 12 hours at 300 °C) nanofluid samples; and (**c**) spectral optical characteristics, and (**d**) hydrodynamic particle size distribution of the as-prepared and after heating (for 72 hours at 300 °C) nanofluid samples (5 ml/L); tested under airtight conditions.

Moreover, nanoparticle size distribution and optical characteristics were retained even after constant heating for 12 hours at 300 °C [see Fig. 8(a,b)]. However, if the heating period is extended to longer duration (72 hours at 300 °C), the particles tend to agglomerate [see Fig. 8(d)]. Although, no particle separation was observed, but some thin deposition on the walls of the container (above the liquid free surface) was observed (due to evaporation and subsequent condensation of the nanofluid on the container surface). This resulted in change in optical properties of the nanofluid [see Fig. 8(c)].

#### Stability under ultra-violet (UV) light exposure

Although ultraviolet radiations form only a small fraction of the incident solar energy; but given the fact that these are very high energy radiations, and may amount to significant values in case of concentrating solar thermal systems - the as-prepared nanofluids were tested exclusively under UV exposure. The nanofluid to be tested was housed in a glass tube and was placed at a distance of 70 mm from the UV light source. The incident flux at the location was measured to be 117 ± 2 Wm^−2^ with the help of a thermopile detector (818P-015-18HP, Newport) and power meter (1918-R, Newport, calibrated at *λ* = 355 nm).

Now, in order to calculate the total exposure energy; Schwarzschild law has been invoked, given by Eq. (3) as

3 $$E=I{t}^{\rho }$$

where *E* is the measured exposed energy, *I* ( = 117 Wm^−2^) is the light source intensity, *t* is the time and *ρ* ( = 0.9) is the Schwarzschild coefficient. Exposed energy (*E*) for 8 hours light exposure amounts to approximately 760 Whm^−2^.

Now, taking E = 760 Whm^−2^ and peak solar UV intensity (AM 1.5) reaching the nanofluid to be 6.5 Wm^−2^; Eq. (3) is invoked to calculate *t*. This comes out to be 198.53 hours (or 24.8 days, with each day of 8 hours sunshine) of peak sunlight, i.e., 8 hours of UV exposure in the UV chamber is equivalent to 24.8 days^30^.

In the present work, the nanofluid sample has been tested for 5 UV cycles. Each cycle consisting of 8 hours of UV exposure, followed by 16 hours of darkness (this is in accordance with the procedure given in ref.^30^). In other words, the nanofluid has been effectively tested for 124 days of sunlight exposure.

**Figure 9:**
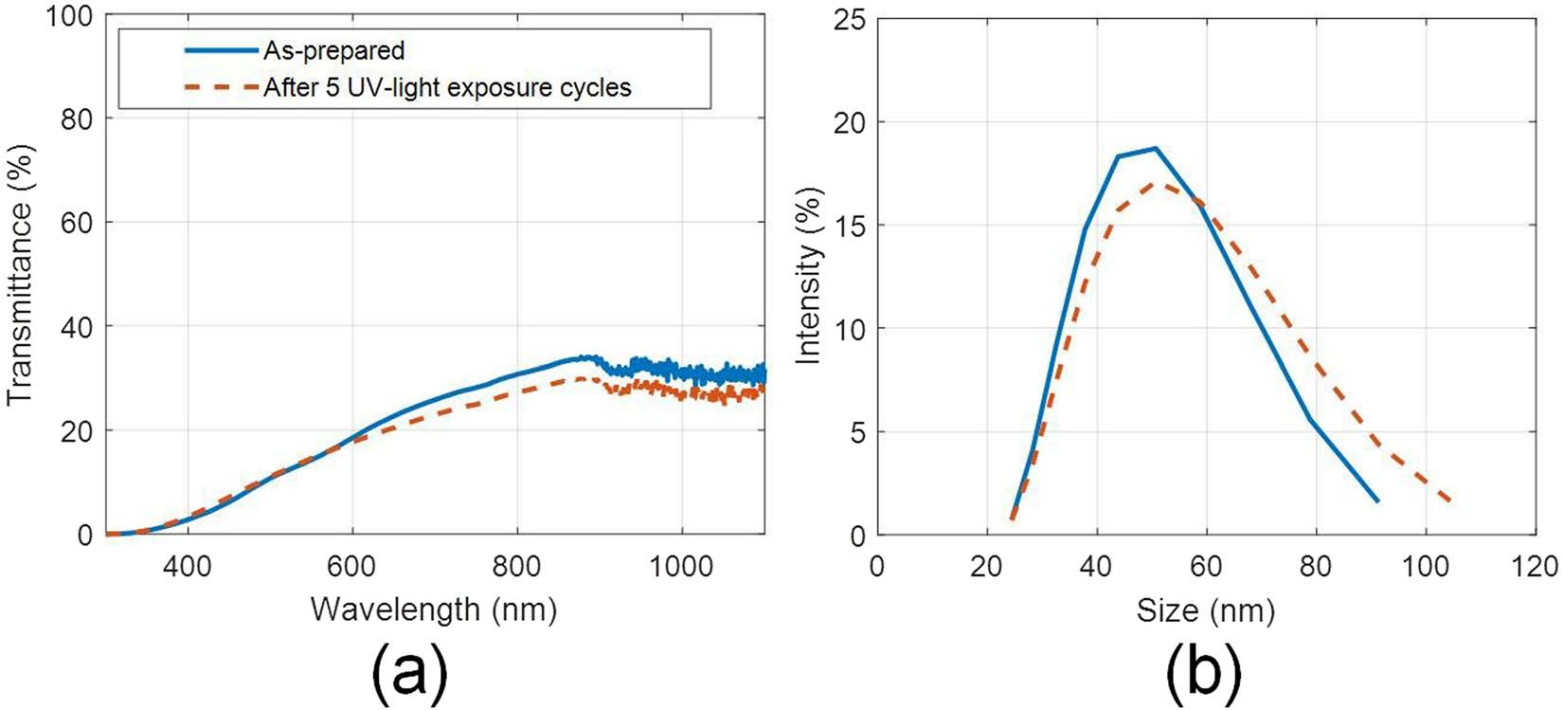
(**a**) Effect of UV exposure (5 cycles) on the (**a**) optical properties, and (**b**) hydrodynamic particle size distribution of the as-prepared nanofluid (5 ml/L).

Interestingly, the nanofluids were found to be stable and retain their properties even after prolonged exposure to UV radiations (see Fig. 9) - as there is only a small change in average particle size (~2%) and also very less change in the optical characteristics. This is a significant improvement, as exposure to UV radiations has been known to significantly impact the stability of the nanofluids, i.e., extensive agglomeration and settling of the untreated nanoparticles occurs when exposed to UV radiations^30^.

### Design and performance of the as-prepared nanofluid based volumetric absorption solar thermal platform

**Figure 10:**
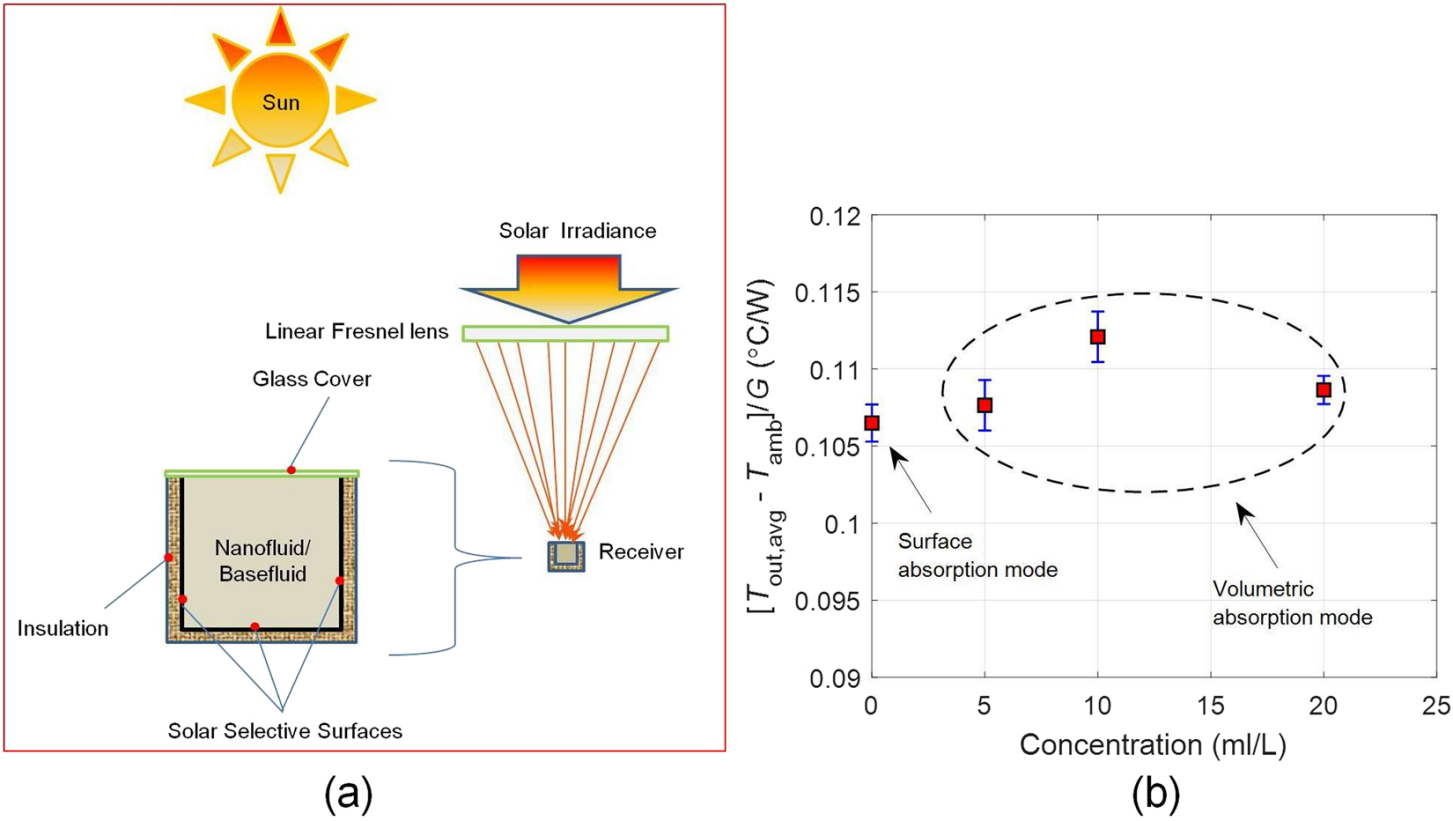
(**a**) Schematic showing the design of the volumetric absorption platform (end view), and (**b**) Steady state temperatures under surface and volumetric (at various nanofluid concentrations) absorption modes. Error bar represents the standard deviation.

Rigorous testing of the as-prepared nanofluids has revealed that indeed these could operate under real world service conditions without losing their functional properties. Building upon this, we have carefully designed a volumetrically absorbing solar thermal system, wherein a linear Fresnel lens is used to concentrate the incident normal solar irradiance onto the receiver lying along the focal line of the concentrator. The receiver is essential a closed rectangular conduit in which the fluid is made to flow. The conduit has been so designed that it could be employed both in surface as well as volumetric absorption modes - the internal three sides (bottom and sides) being ‘state of the art’ solar selective surfaces (having high solar weighted absorptivity, ~0.96; and low infrared emissivity, ~0.12) and the top side being made of glass to allow the sunlight to pass through and reduce thermal losses. Furthermore, the outside three sides have been insulated to reduce thermal losses [see Fig. 10(a)].

In surface absorption mode, pure paraffin oil was made to flow in the receiver. Paraffin oil being nearly transparent in the solar wavelength region, allows the sunlight to interact with the solar selective surfaces. The absorbed solar energy is then transferred to the fluid through convection and conduction mechanisms. This configuration essentially simulates the heat transfer mechanisms involved in typical incumbent solar thermal systems.

On the other hand, in volumetric absorption mode, the as-prepared nanofluids (of different concentrations) were made to flow through the receiver. Herein, the nanofluid directly absorbs the solar energy - nanoparticles owing to their broad wavelength absorption characteristics; convert the solar energy into thermal energy through non-radiative decay of the absorbed photons. Subsequently, the absorbed energy is transferred to the surrounding medium at staggeringly rapid rate (owing to the extremely small size of the particles). This results in efficient photo-thermal conversion of the incident solar energy.

Comparison between the two absorption modes (surface and volumetric) clearly points out that indeed higher (~5% higher) steady state temperatures [averaged over the receiver depth, see Supplementary Fig. S8] could be achieved in case the sunlight is allowed to directly interact with the nanofluid i.e., volumetric absorption outperforms the surface absorption case under real world flow conditions and under the sun [see Fig. 10(b)]. Furthermore, careful observation of the graphs tell us that it is imperative to choose the right concentration of the nanofluids (for a given fluid layer thickness) in order to maximize the resulting steady state temperatures and hence the performance characteristics.

## Conclusions

As a whole, the present work reports a simple, cost effective and scalable method to synthesize broad absorption nanofluids from ‘used engine oil’. These as-prepared nanofluids have shown to possess remarkable properties in relation to its candidature as a potential working fluid in volumetric absorption solar thermal systems. Building on it, an efficient solar thermal platform has been devised which gives higher steady state temperatures in volumetric absorption mode. Thus, we have been able to engineer efficient volumetric absorption solar thermal platforms which outperform their surface absorption counterparts (conventional solar thermal systems) under realistic conditions and under the sun.

## Methods

### Nanofluid preparation

Used engine oil has been collected from a 15000 km run 4-stroke diesel engine. To filter out sludge, resin etc. a cotton cloth has been used. Subsequently, the filtered ‘used engine oil’ was furthered filtered with 0.7 μm filter paper. Desired fractions of the resulting filtered used oil were then mixed into pure paraffin oil followed by 30 minutes of ultra-sonication in a bath type ultasonicator - thus forming nanofluids of different concentrations.

### Characterization and measurements

EDS. Used engine oil cannot be directly analyzed by EDS because of the presence of oil which may contaminate the electron beam. So sample was prepared by evaporating 20 ml/liter sample at 160°C and the leftover after evaporation was collected. The collected solid particles were then washed with the ethanol (5 times) in order to remove any traces of oil. Finally, the washed particles were loaded onto the copper grid for EDS analysis.

Spectral analysis. Transmittance measurements in the UV-VIS-NIR region were done using spectrophotometer. Shimazdu UV-2600.

Fourier transform infrared (FTIR) spectroscopy. Infrared measurements were made using attenuated total reflectance (ATR) technique with help of Nicolet iS50 FT-IR.

TGA. Nitrogen atmosphere, 30°C to 800°C at a heating rate of 10°C/min, TGA/DSC 1 - Thermogravimetric Analyzer, Mettler Toledo.

TEM. The sample for TEM analysis was prepared by solvent extraction method. The used oil was mixed with n-heptane (1:60) and ultra-sonicated for 30 minutes. The prepared sample was then placed on the carbon grid and washed with n-heptane to remove any traces of oil on the surface of soot particles. Furthermore, to get the better image quality, sample was washed with diethyl ether (2 times). TEM, FEI Tecnai G2 F20, Netherlands.

DLS. Hydrodynamic size distribution measurements were made using Malvern Zetasizer Nano S (ZEN 1600).

Thermal conductivity of the as-prepared nanofluids was measured by KD2 pro which works on the transient hot wire method.

Raman Spectra. Measured at 532 nm, Horiba Scientific.

Viscosity measurements were made using capillary action viscometer.

White light source. Light guide connected to a 3200 K Color temperature halogen lamp (250 W), Philips.

UV exposure tests. UV exposure tests have been done in a custom designed ultraviolet light chamber (photo-chemical reactor). The UV light source being a 125 W (HPL-N 125 W E27, Philips) mercury vapor lamp (outer phosphorous coated cover removed) surrounded with water jacket to maintain a constant temperature of the lamp.

Temperature measurements. K type thermocouples and infrared camera, Keysight.

Data Acquisition (DAQ). DAQ card NI 9123, Chassis NI 9721.

Incident solar flux, direct normal irradiance (DNI). Ring shaded Pyranometer, Kipp & Zonen.

Heating sources. Metal top and ceramic based hot plates.

Ultra-sonicator. Bath type − 250 W, Sarthak Scientific.

Error bar represents the standard deviation and is given by $\sigma =\sqrt{\frac{\sum _{i=1}^{n}{(\overline{x}-{x}_{i})}^{2}}{n}}$.

### Constructional and operational parameters of the volumetric absorption solar thermal platform

Linear Fresnel lens. 2 in number, each of length 500 mm, width 500 mm PMMA-3t, NTKJ, Japan.

Solar selective surface. Black chrome coated copper sheet, Solchrome.

Dimensions of the receiver (internal). length 1000 mm, width 29 mm, and height 20 mm.

Volume flow rate. 0.5 lpm.

## Supplementary information

Supplementary Information
