# Supplementary material for: Efficient Volumetric Absorption Solar Thermal Platforms Employing Thermally Stable - Solar Selective Nanofluids Engineered from Used Engine Oil
Source: Sci Rep. 2019 Jul 22;9:10541. doi: 10.1038/s41598-019-47126-3 (PMC6646354; doi:10.1038/s41598-019-47126-3)
Supplement: Supplementary file 1 — Supplementary Information [file 41598_2019_47126_MOESM1_ESM.pdf]

Efficient Volumetric Absorption Solar Thermal Platforms Employing Thermally Stable - Solar Selective Nanofluids Engineered from 'Used Engine Oil'

Nirmal Singh and Vikrant Khullar*

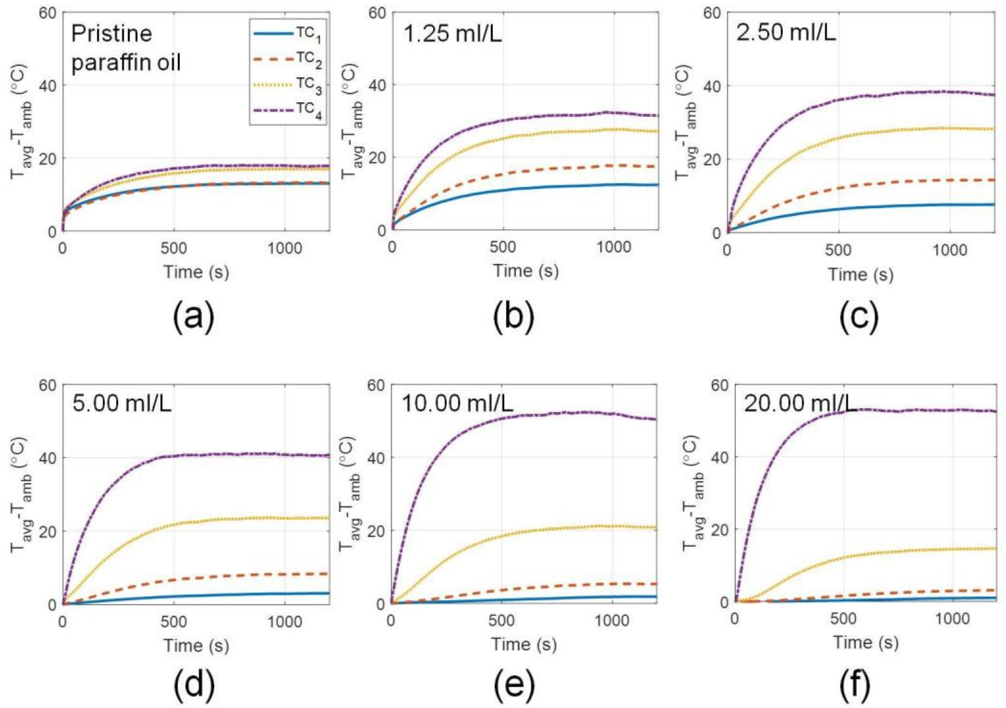

Fig. S1 Spatial temperature distribution, when the as-prepared nanofluids are illuminated under a white light source. (a) Pristine paraffin oil, (b) 1.25 ml/L, (c) 2.50 ml/L, (d) 5.00 ml/L, (e) 10.00 ml/L, and (f) 20.00 ml/L.

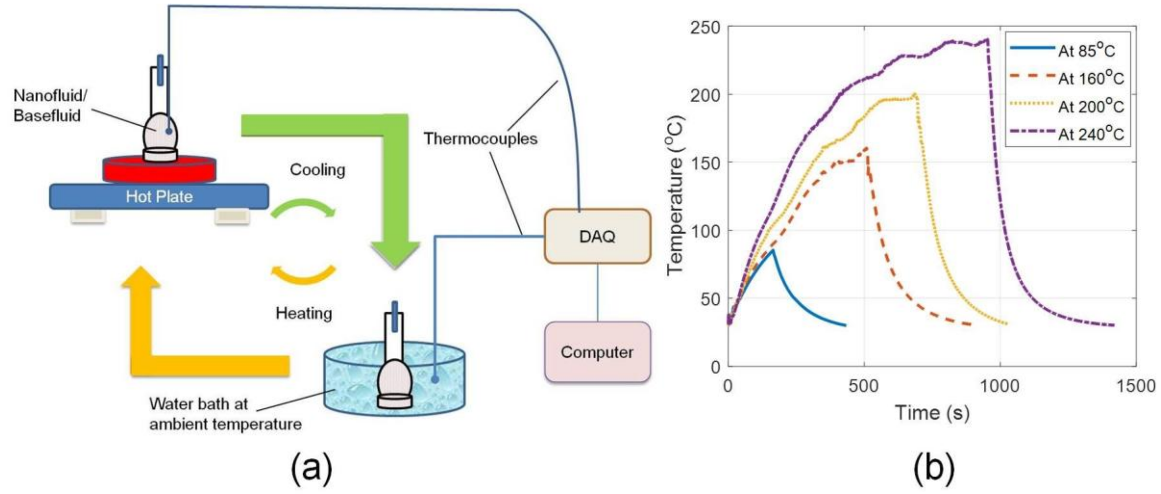

Fig. S2 (a) Schematic showing the procedure followed for carrying out thermal cyclic tests, and (b) heating - cooling curves for the thermal cycling at different temperatures.

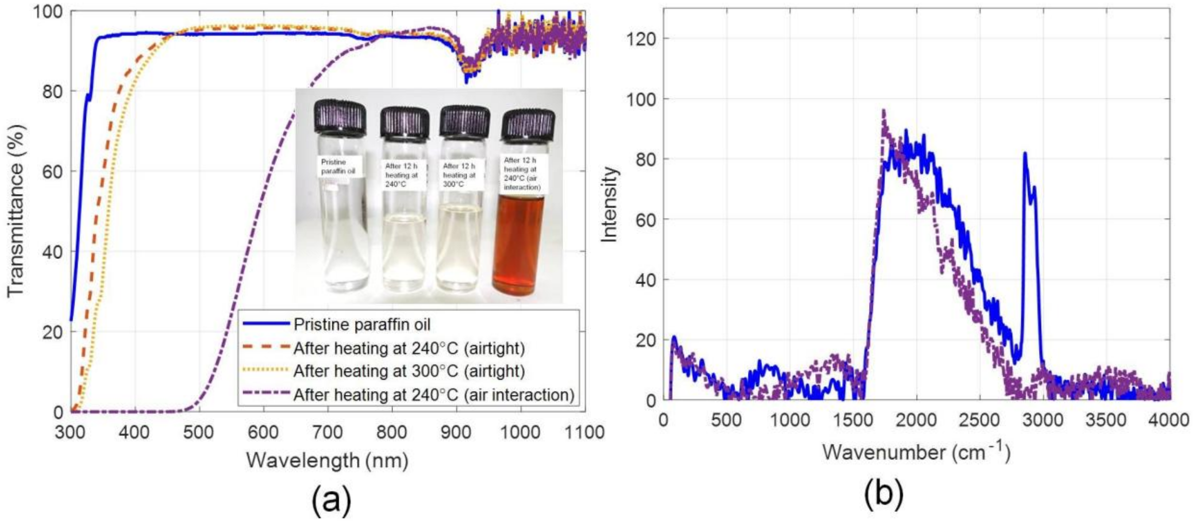

Fig. S3 (a) Effect of oxidation on the (a) optical properties, and (b) Raman spectra of pristine paraffin oil.

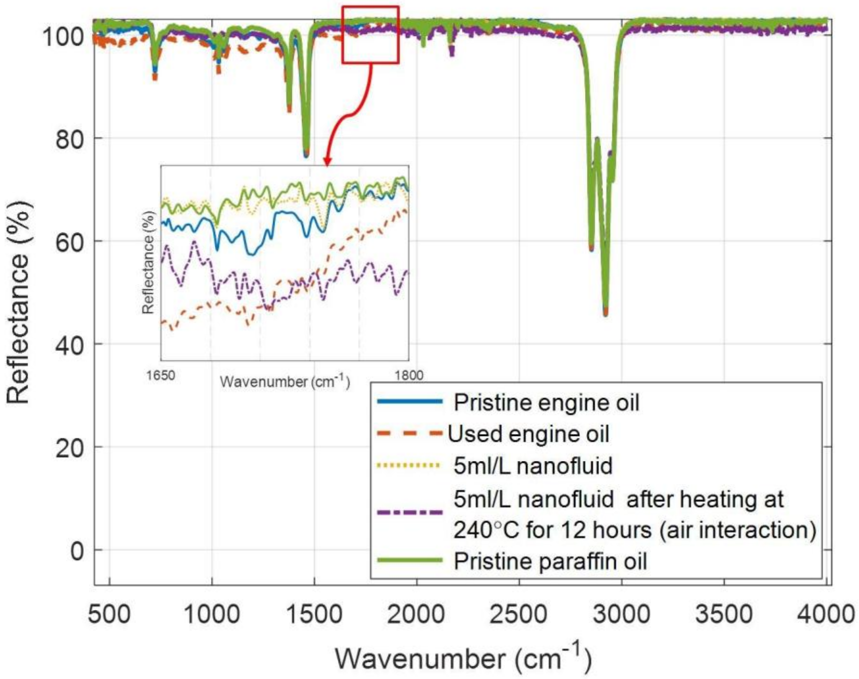

Fig. S4 ATR-FTIR spectra showing the effect of oxidation on nanofluid (5ml/L); 1650 cm^{-1} - 1800 cm^{-1} being the region of oxidation products[1]. For better comparison; spectra has also been shown for pristine engine oil, used engine oil and pristine paraffin oil.

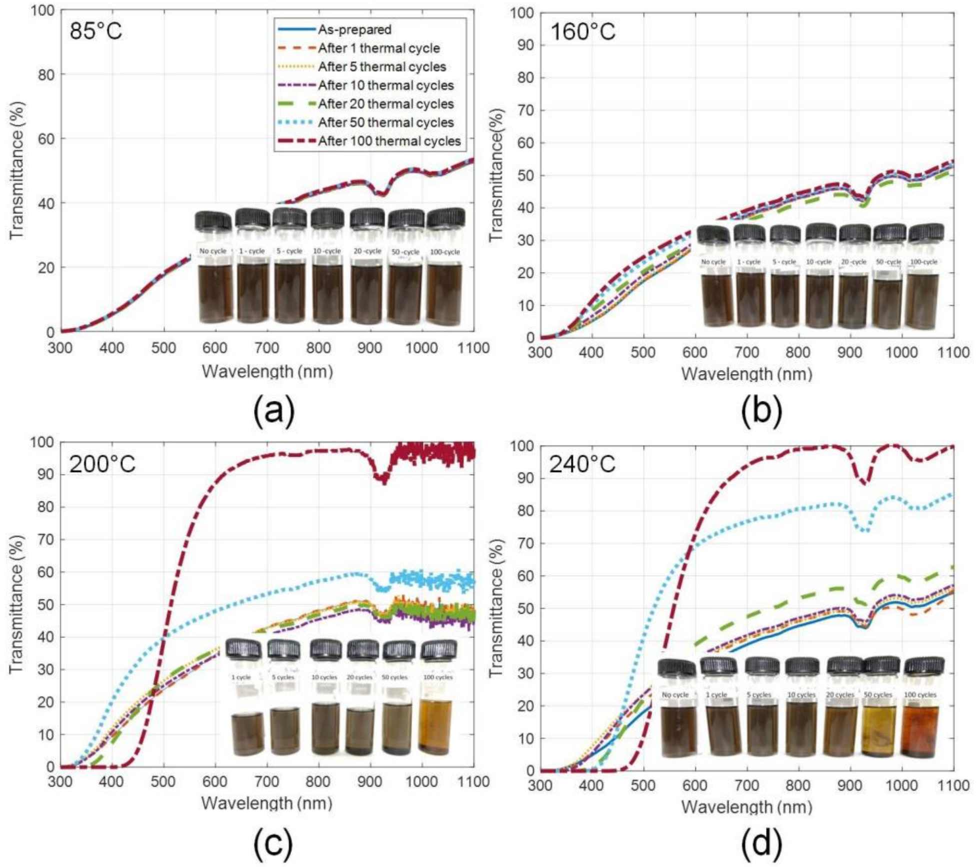

Fig. S5 (a) Effect of cyclic thermal loads on the optical properties of the as-prepared nanofluids at (a) 85°C, (b) 160°C, (c) 200°C, and 240°C.

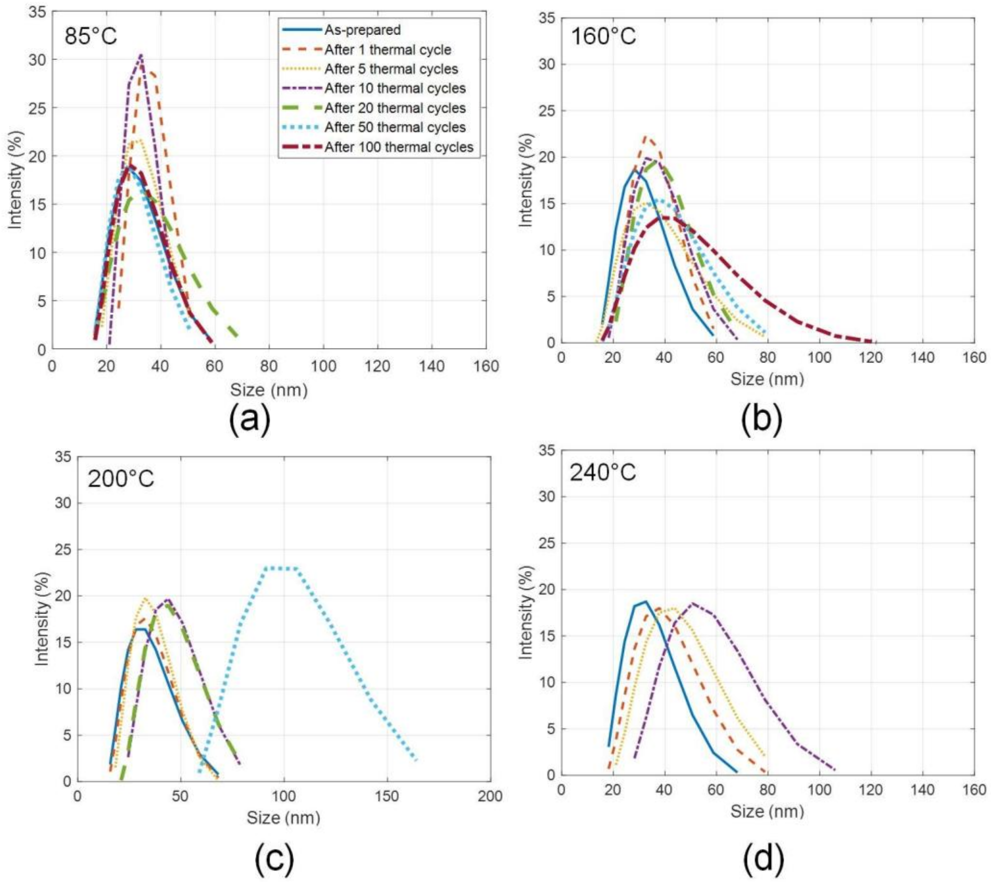

Fig. S6 (a) Effect of cyclic thermal loads on the hydrodynamic size distribution of the as-prepared nanofluids at (a) 85°C, (b) 160°C, (c) 200°C, and 240°C.

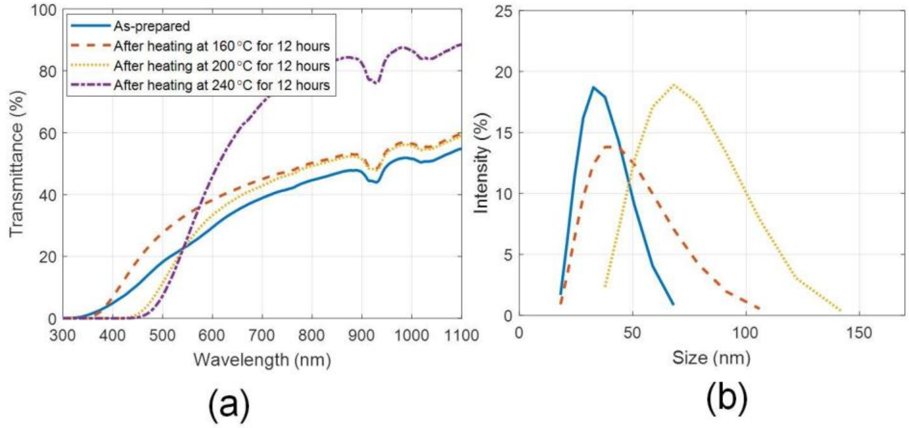

Fig. S7 (a) Effect of prolonged heating (for 12 hours) on the (a) optical properties, and (b) hydrodynamic size distribution of the as-prepared nanofluid (5 ml/L).

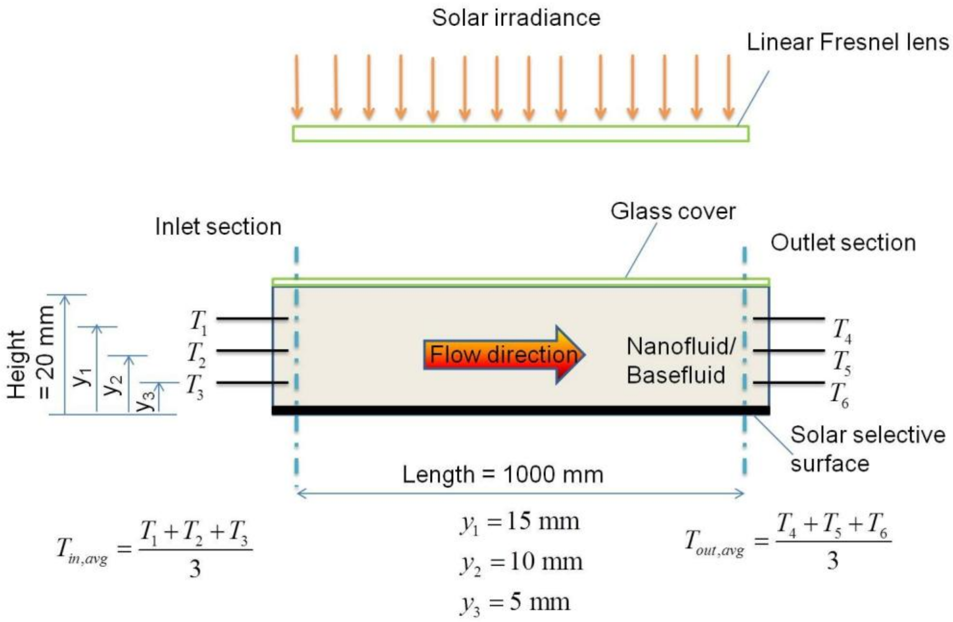

Fig. S8 Schematic showing the longitudinal view of the receiver. Calculation of inlet and outlet temperatures averaged over the depth direction.

References

1. Ahmad, I. *et al.* Monitoring of oxidation behavior in mineral base oil additized with biomass der ived antioxidantsusing FT-IR spectroscopy. *RSC Adv.* **5**, 101089–101100 (2015).
